# Long non-coding RNA XIST regulates gastric cancer progression by acting as a molecular sponge of miR-101 to modulate EZH2 expression

**DOI:** 10.1186/s13046-016-0420-1

**Published:** 2016-09-13

**Authors:** Dong-liang Chen, Huai-qiang Ju, Yun-xin Lu, Le-zong Chen, Zhao-lei Zeng, Dong-sheng Zhang, Hui-yan Luo, Feng Wang, Miao-zhen Qiu, De-shen Wang, Da-zhi Xu, Zhi-wei Zhou, Helene Pelicano, Peng Huang, Dan Xie, Feng-hua Wang, Yu-hong Li, Rui-hua Xu

**Affiliations:** 1 State Key Laboratory of Oncology in South China, Collaborative Innovation Center for Cancer Medicine, Department of Medical Oncology, Sun Yat-sen University Cancer Center, No. 651 Dong Feng East Road, Guangzhou, 510060 China; 2 University of Texas M.D. Anderson Cancer Center, Houston, TX USA

**Keywords:** Long non-coding RNA, lncRNA XIST, miR-101, EZH2, Gastric cancer

## Abstract

**Background:**

Long non-coding RNAs (lncRNAs) have emerged as critical regulators of tumor progression. However, the role and molecular mechanism of lncRNA XIST in gastric cancer is still unknown.

**Methods:**

Real-time PCR analysis was performed to measure the expression levels of lncRNA XIST in gastric cancer tissues and cell lines, the correlation between lncRNA XIST expression and clinicopathological characteristics and prognosis was analyzed in gastric cancer patients. The biological function of lncRNA XIST on gastric cancer cells were determined both in vitro and in vivo. The regulating relationship between lncRNA XIST and miR-101 was investigated in gastric cancer cells.

**Results:**

lncRNA XIST was significantly up-regulated in gastric cancer tissues and cell lines. Overexpression of lncRNA XIST was markedly associated with larger tumor size, lymph node invasion, distant metastasis and TNM stage in gastric cancer patients. Functionally, knockdown of lncRNA XIST exerted tumor-suppressive effects by inhibiting cell proliferation, migration and invasion in vitro and tumor growth and metastasis in vivo. Furthermore, an inverse relationship between lncRNA XIST and miR-101 was found. Polycomb group protein enhancer of zeste homolog 2 (EZH2), a direct target of miR-101, could mediated the biological effects that lncRNA XIST exerted.

**Conclusions:**

lncRNA XIST is up-regulated and is associated with aggressive tumor phenotypes and patient survival in gastric cancer, and the newly identified lncRNA XIST/miR-101/EZH2 axis could be a potential biomarkers or therapeutic targets for gastric cancer patients.

## Background

Gastric cancer is one of the most common malignant diseases and the second leading cause of cancer-related mortalities worldwide [1]. Despite great developments in the diagnosis and therapy of this disease in the past decades, the overall survival rate of gastric cancer patients is still unsatisfied. In most cases, gastric cancer is diagnosed at advanced stage which is characterized with malignant proliferation, extensive invasion and distant metastasis. Traditionally, the TNM stage was used as an indicator to predict prognosis of patients, recent studies have proved that the criteria alone is not sufficient for estimating prognosis [2, 3]. Therefore, there is an urgent need to identify novel biomarkers that can predict patient survival and be used as therapeutic targets. Previously, we have found that L1cam plays a critical role in the progression of gastric cancer and Paxillin is a prognostic indicator of gastric cancer patients [4, 5]. Recent years, evidences have indicated that long non-coding RNAs (lncRNAs) also act as modulators in the progression of gastric cancer and might serve as novel therapeutic targets [6].

As the development of the human genome project, it has been recognized that the vast majority of mammalian genome are transcribed to produce non-coding RNAs (ncRNAs) [7]. Among which are a new group of RNAs, known as long non-coding RNAs (lncRNAs). lncRNAs are a class of transcripts which are greater than 200 nt in size and lack significant protein-coding capacity. lncRNAs are functionally diverse which can act as guides, decoys, scaffolds and tethers of other biological molecules [8]. Recent studies indicated that lncRNAs could competitively suppress miRNAs by acting as molecular sponges [9]. For instance, it has been found that lincRNA-ROR acted as a molecular sponge for miR-145 in triple-negative breast cancer [10]. lncRNA NEAT1 promotes laryngeal squamous cell cancer through regulating miR-107/CDK6 pathway [11]. Increasing evidences demonstrated that lncRNAs are critical regulators of multiple biological processes, including cell growth, cell apoptosis, cell differentiation, cell invasion and stem cell pluripotency [12–16].

The lncRNA XIST (X-inactive specific transcript) is a product of the XIST gene and the master regulator of X inactivation in mammals [17]. More and more studies indicated that lncRNA XIST plays critical role in cell proliferation, differentiation, and genome maintenance. It was found that lncRNA XIST is dysregulated in different cancers. For instance, dysregulation of lncRNA XIST may leads to alterlation of gene expression and instability of heterochromatin [18]. lncRNA XIST was essential for long term survival of hematopoietic stem cells [19]. A recent study demonstrated that knockdown of lncRNA XIST exerted tumor-suppressive effects in human glioblastoma stem cells through up-regulating miR-152 [20]. However, the expression and biological function of lncRNA XIST in gastric cancer is unclear.

Polycomb group protein enhancer of zeste homolog 2 (EZH2) is a methyltransferase and the core catalytic element of polycomb repressive complex 2, which plays a critical role in the regulation of cell proliferation, migration, invasion, tumorigenesis and metastasis [21, 22]. EZH2 has been found to be involved in multiple tumors, including gastric cancer [23–25]. Mastukawa and his colleagues were the first to report the role of EZH2 and its prognostic significance in gastric cancer [24]. More recently, it has been shown that up-regulation of EZH2 contributes to gastric cancer invasion and metastasis [26, 27].

In this study, we found that lncRNA XIST expression was significantly up-regulated in gastric cancer tissues and cell lines and affected clinicopathological characteristics and prognosis in gastric cancer patients. Moreover, knockdown of lncRNA XIST could inhibit gastric cancer cell proliferation and invasion in vitro as well as tumorigenesis and metastasis in vivo. Based on a bioinformatic analysis, we found lncRNA XIST could act as a molecular sponge of miR-101. Furthermore, knockdown of lncRNA XIST exerts its tumor-suppressive effect though down-regulating the expression of EZH2 via miR-101. Our study provides the first evidence of the regulatory mechanisms of the newly identified lncRNA XIST/miR-101/EZH2 axis in carcinogenesis and metastasis, which may shed light on their targeted applications in cancer therapies.

## Methods

### Human tissue specimens and cell culture

Fresh-frozen cancer tissues and paired normal gastric epithelial tissues were obtained from 106 patients who undergoing surgery at the Sun Yat-sen University Cancer Center from 2008 to 2010. The study was approved by the ethics committee of the Sun Yat-sen University Cancer Center and informed consent was obtained from all patients. All the patients did not receive any treatment before the operation. Each patient was followed up regularly every 3 months after surgery. The clinicopathological characteristics including age, gender, tumor size, differentiation, lymph node invasion, peritoneum dissemination, distant metastasis and TNM stage were recorded. Overall survival was defined as the time from the date of surgery to the date of death or last contact.

Human embryonic kidney (HEK) 293 T cells, human gastric cancer cell lines (SGC7901, HGC27, BGC823, MKN45, MKN28, and AGS) and the normal gastric epithelial cell line GES-1 were obtained from either the type Culture Collection of Chinese Academy of Sciences (Shanghai, China) or the American Type Culture Collection. These cells were cultured and stored according to the provider’s instructions. Cells were routinely authenticated every 6 months (last examined in January 2016) by cell morphology monitoring, growth curve analysis and testing for mycoplasma (R&D Systems’ new MycoProbe Mycoplasma Detection Kit).

### RNA extraction and real-time PCR analysis

Total RNA was extracted from tissue samples and cells using Trizol reagent (Invitrogen, Carlsbad, CA) according to the manufacturer’s protocol. High Capacity cDNA Reverse Transcription Kit (Applied Biosystems, Foster City, CA) was used for lncRNA XIST and EZH2 reverse transcription. Quantitative real-tme PCR was performed using TaqMan Universal Master Mix II, and GAPDH was used as the internal control. The following primers were used for the quantitative PCR:

- lncRNA XIST
- Forward: 5'-CTCTCCATTGGGTTCAC-3';
- Reverse: 5'-GCGGCAGGTCTTAAGAGATGAG-3';
- EZH2
- Forward: 5'-TGCAGTTGCTTCAGTACCCATAAT-3';
- Reverse: 5'-ATCCCCGTGTACTTTCCCATCATAAT-3';
- GAPDH
- Forward: 5'-TGCACCACCAACTGCTTAGC-3';
- Reverse: 5'-GGCATGGACTGTGGTCATGAG-3'.

For the measurement of miR-101, the All-in-One™ miRNA qRT-PCR Detection Kit (GeneCopoeia) was used according to the manufacturer’s instructions; U6 small RNA was used as the reference. Real-time PCR was performed with the Bio-Rad CFX96 qPCR system, and fold changes were determined using the relative quantification 2^-△△CT^ method.

### Lentivirus production and infection

Short hairpin RNA (shRNA) directed against human lncRNA XIST or scrambled oligonucleotides were ligated into the LV-3 (pGLVH1/GFP + Puro) vector (GenePharma, Shanghai, China). The HEK293 cells were cotransfected with Lenti-Pac HIV Expression Packaging Mix and the lentiviral vectors (or the control lentivirus vectors) using Lipofectamine 2000 (Life Technologies Corporation, Carlsbad, CA, USA). After 48 h, lentiviral particles in the supernatant were harvested and filtered by centrifugation at 500 g for 10 min. The SGC7901 and AGS cells were then transfected with Lentivirus or control virus (NC). In order to select the stably transfected cells, the cells were treated with puromycin (2 μg/ml) for 2 weeks. GFP-positive cells were picked as sh-XIST and sh-NC, and then used for subsequent assays.

### Cell transfections

Cell transfections were performed using Lipofectamine 2000 (Invitrogen, Carlsbad, CA, USA) according to the provider’s instructions. The hsa-miR-101 mimic, has-miR-101 inhibitor and negative control (NC) oligonucleotides were obtained from Ribobio (Guangzhou, China). To restore EZH2 expression, the SGC7901 cells were contransfected with has-miR-101 minics and a pcDNA3.1-EZH2 plasmid, which contained the coding sequences but lacked the 3′-UTR of EZH2. The cells were plated in a 6-well plate the day before transfection. The cells were transfected with a final concentration of 50 nM and collected for assays after 48 h.

### Cell proliferation assays

Cell proliferation ability was determined by performing CCK-8 assay and the colony formation assay. For CCK-8 assay, cells were seeded into a 96-well plate and cultured at 37 °C. Each well was added with 10 μl CCK-8 solution. Then, plates were incubated at 37 °C for 2 h. Finally, the spectrophotometric absorbance at 570 nm was measured for each sample. All the experiments were repeated 3 times in triplicate and the mean was calculated. For the colony formation assay, 2000 cells were placed in a six-well plate and cultured with RPMI 1640 medium (GIBCO) containing 10 % FBS for 2 weeks. Colonies were fixed with methanol and stained with 0.1 % crystal violet (1 mg/ml).

### In vitro invasion and migration assay

The cell invasive and migratory potential was evaluated using transwell and wound healing assays, respectively. The cell invasive potential was determined using transwell assay. Briefly, the cells were suspended in serum-free medium and seeded in the top chamber (8-μm pore; BD Biosciences) of the inserts. 500 μl of FBS was added to the lower chamber as the chemoattractant. 22 h later, the non-migrated cells on the top surface were swabbed off gently, and the cells invaded to the lower compartment were fixed with methanol, and stained with 0.1 % crystal violet. The number of invaded cells was calculated by counting five random views under the microscope. The experiment was performed in triplicate and repeated for three times. For wound healing assay, the cells were seeded in 6-well plates, and an artificial wound was created using a 200-μl pipette tube. The wound closure was observed after 36 h and imaged under a microscope. We measured the fraction of cell coverage across the line for the migration rate.

### In vivo tumorigenesis and metastasis assays

All the animal experiments were performed according to the National Institutes of Health animal use guidelines on the use of experimental animals. Female BABL/c athymic nude mice (4 to 5 weeks old) were obtained from the Animal Center of Guangdong province (Guangzhou, China). To evaluate the in vivo tumorigenesis effect of lncRNA XIST, the SGC7901/sh-NC and SGC7901/sh-XIST cells (1 × 10^6^cells/mouse) were injected subcutaneously into the flanks of two groups of nude mice (ten for each cell group). Tumor size was measured every 4 days and tumor volume was estimated. After 5 weeks, the mice were killed and the tumors were dissected out. To investigate the effect of lncRNA XIST on tumor metastasis, the SGC7901/sh-NC and SGC7901/sh-XIST cells (2 × 10^6^cells/mouse) were injected into the tail vein of two groups of nude mice (ten for each cell group). Six weeks post injection, the mice were killed and the livers were removed and paraffin embedded. Consecutive sections (4 μm) were made and stained with haematoxylin-eosin. The micro-metastases in the livers were examined and counted under a dissecting microscope.

### Reporter vector construction and luciferase reporter assay

We used the bioinformatics databases (Starbase v2.0, miRcode and RNAhybrid) to search for potential microRNAs that can bind to lncRNA XIST. The fragment from lncRNA XIST containing the predicted miR-101 binding site was amplified by PCR and cloned into a pmirGLO Dual-luciferase Target Expression Vector (Promega, Madison, WI, USA) to form the reporter vector XIST-wild-type (pmirGLO-XIST-Wt). To test the binding specificity, the corresponding mutant was created by mutating the miR-101 seed region binding site (seed sequence binding fragment 5′-GCACTG-3′ changed to 5′-AAGTGA-3′), which were named as pmirGLO-XIST-Mt. HEK 293 T cells were co-transfected with the pmirGLO vector with either wild type fragments or mutation fragments and indicated miRNAs using Lipofectamine 2000. Luciferase reporter assay was performed using the Dual-Luciferase Reporter Assay System (Promega, Madison, WI, USA) 48 h later, the firefly luciferase activity was measured and normalized by renilla luciferase activity. To confirm the direct regulating relationship between miR-101 and EZH2, the full-length 3′-UTR of the EZH2 mRNA and a mutant variant were amplified by PCR and cloned into the XbaI site of a pGL3-basic vector (Promega) and termed EZH2-3′UTR and EZH2-mt-3′UTR, respectively. The miR-101 expression plasmid (pcDNA-miR-101) was generated using synthetic oligonucleotides and the pcDNA6.2-GW/EmGFP vector. Cells were cultured in a six-well plate and then transfected with the pcDNAmiR-101 or the negative control (NC) (750 ng/well), the pGL3 reporter vector (250 ng/well) and the pRL-TK luciferase reporters (25 ng/well) using Lipofectamine 2000 (Invitrogen). Luciferase activity levels were measured using the Dual-Luciferase Reporter Assay Kit (Promega) following the manufacturer’s instructions.

### Western blot analyses

Total proteins were extracted from tissues or cells and separated using SDS-PAGE gels. Antibodies for EZH2, E-cadherin, α-catenin, Vimentin and Fibronectin were purchased from Cell Signaling Technology, and an anti-GAPDH antibody (1:2000; Santa Cruz Biotechnology, USA) was used as a loading control. The procedure of Western blot analysis was performed as previously described [4].

### Immunohistochemistry (IHC) analysis

The paraffin-embedded tissue blocks were cut into 4 μm slides. Rabbit mmp-9 antibody and rabbit Ki-67 antibody purchased for Cell Signaling Technology. IHC analysis was performed according to a previously described method [4].

### Statistical analyses

Statistical analyses were performed using the SPSS software package (version 16.0, SPSS Inc.) or GraphPad Prism 5.0. Survival curves were generated using the Kaplan-Meier method and assessed using the log-rank test. The Cox proportional hazard regression model was performed to identify independent prognostic factors. *P* < 0.05 was considered to be statistically significant.

## Results

### lncRNA XIST expression is up-regulated in gastric cancer tissues and cell lines

**Fig. 1:**
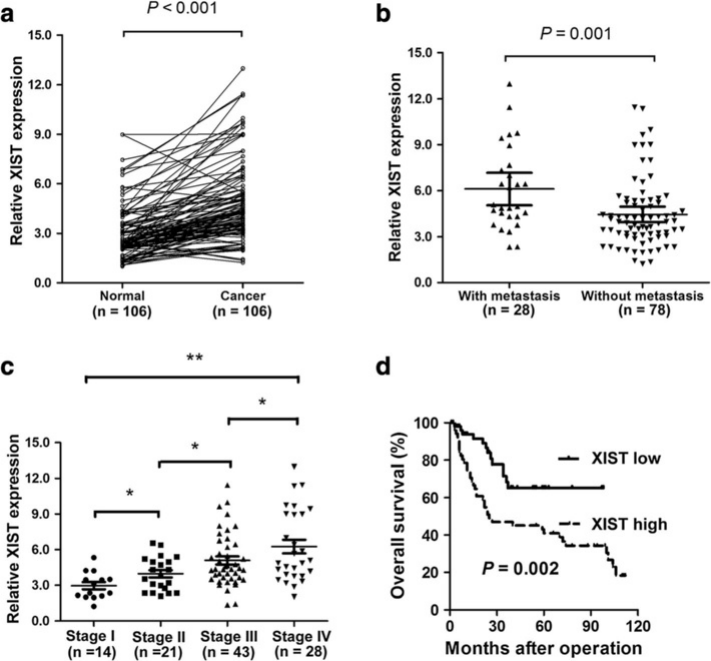
lncRNA XIST is significantly up-regulated in gastric cancer tissues and cell lines. **a** Relative expression level of lncRNA XIST in gastric cancer tissues (*n* = 106) and adjacent normal tissues, lncRNA XIST expression was significantly higher in gastric cancer tissues as compared with adjacent normal tissues (*n* = 106) (*P* < 0.001). **b** Relative expression level of lncRNA XIST in gastric cancer tissues with (*n* = 28) and without distant metastasis, lncRNA XIST expression was lower in tissues without distant metastasis compared with tissues with distant metastasis (*n* = 78) (*P* = 0.001). **c** Relative expression level of lncRNA XIST in different clinical stage (*P < 0.05, **P < 0.001). **d** Kaplan-Meier curve of overall-survival in gastric cancer patients with high lncRNA XIST level (*n* = 54) and low lncRNA XIST level (*n* = 52), the overall survival time was shorter in patients with high lncRNA XIST than those with low lncRNA XIST (*P* = 0.002)

**Fig. 2:**
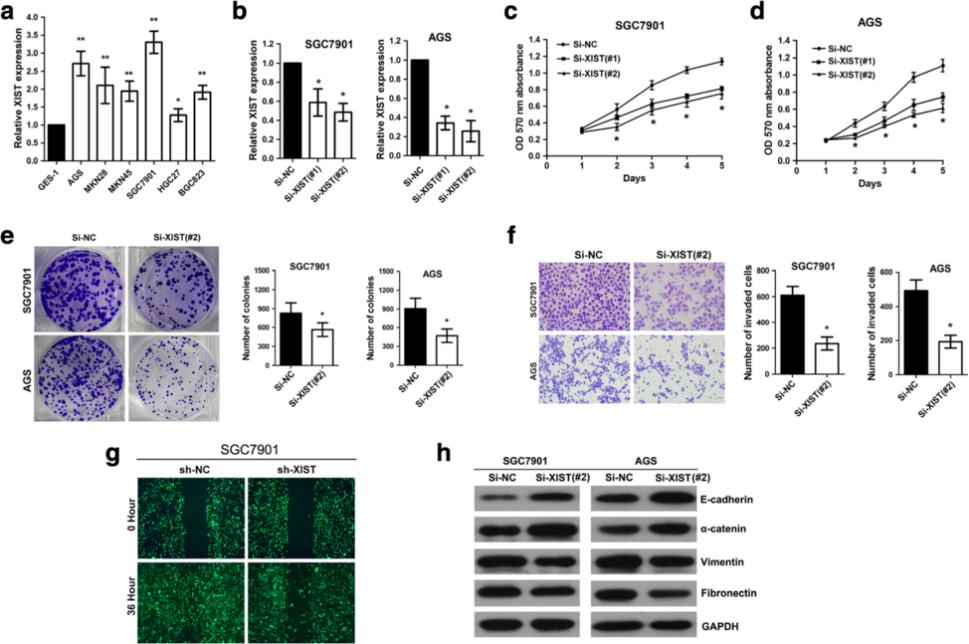
Knockdown of lncRNA XIST expression inhibits gastric cancer cell growth, colony formation, migration and invasion *in vitro*. **a** Relative expression level of lncRNA XIST in different gastric cancer cell lines (*P < 0.05, **P < 0.001). **b** Real-time PCR confirmed the knockdown of lncRNA XIST in gastric cancer cell SGC7901 and AGS (*P < 0.05). **c** and **d** Knockdown of lncRNA XIST inhibited cell proliferation as indicated by CCK-8 assays in SGC7901 and AGS (One-way ANOVA test, *P < 0.05). **e** Knockdown of lncRNA XIST inhibited colony formation as demonstrated by colony formation assays in SGC7901 and AGS (*P < 0.05). **f** and **g** Knockdown of lncRNA XIST inhibited cell invasion and migration as indicated by transwell and wound healing assays (*P < 0.05). **h** Knockdown of lncRNA XIST increased the level of epithelial markers such as E-cadherin, α-catenin while reduced the level of mesenchymal markers such as Vimentin and Fibronectin in SGC7901 cells

To determine whether lncRNA XIST is associated with gastric cancer development, we first measured the expression level of lncRNA XIST in gastric cancer tissues. The results showed that lncRNA XIST expression was significantly increased in gastric cancer tissues as compared with adjacent normal tissues (Fig. 1a, *P* < 0.001). Higher lncRNA XIST expression was observed in tissues with distant metastasis than tissues without distant metastasis (Fig. 1b, *P* = 0.001). In addition, the lncRNA XIST expression level was markedly correlated with the TNM stage of gastric cancer patients (Fig. 1c, *P < 0.05, **P < 0.001). lncRNA XIST level was then determined in gastric cancer cell lines by real-time PCR analysis. lncRNA XIST level was significantly higher in gastric cancer cell lines (SGC7901, HGC27, BGC823, MKN45, MKN28, AGS) than that of normal gastric epithelial cell GES-1 (*P < 0.05, **P < 0.001, Fig. 2a). These results indicated that increased lncRNA XIST expression might be critical involved in gastric cancer progression.

### lncRNA XIST level is associated with aggressive tumor phenotypes and adverse prognosis in gastric cancer patients

To investigate the clinicopathological role of lncRNA XIST in gastric cancer, The median level of lncRNA XIST expression (4.32) was used as a cutoff value to divide all 106 patients into two groups. Gastric cancer patients who express lncRNA XIST at levels higher than the cutoff value were assigned to the high expression group (*n* = 54, lncRNA XIST expression level ≥ cutoff point), and those with expression lower than the cutoff value were assigned to the low expression group (*n* = 52, lncRNA XIST expression level < cutoff point). The relationship between lncRNA XIST expression level and clinicopathological parameters was summarized in Table 1. High lncRNA XIST expression was significantly associated with larger tumor size (*P* = 0.023), lymph node invasion (*P* = 0.013), distant metastasis (*P* = 0.011) and TNM stage (*P* = 0.016). However, no correlation was observed between lncRNA XIST expression level and age, gender, peritoneum dissemination and differentiation. In addition, Kaplan-Meier analysis demonstrated that patients with high lncRNA XIST expression tended to have worse overall survival than patients with low lncRNA XIST expression (*P* = 0.002, Fig. 1d). Moreover, to evaluate whether the expression of lncRNA XIST was an independent prognostic factor for gastric cancer, univariate and multivariate analyses were performed. Univariate analysis demonstrated that distant metastasis (*P* = 0.018), TNM stage (*P* = 0.027) and XIST expression level (*P* = 0.002) were significantly associated with overall survival of gastric cancer patients (Table 2). However, multivariate analysis using the Cox proportional hazards model for all variables that were significant in the univariate analysis showed that only distant metastasis (*P* = 0.033) and lncRNA XIST expression level (*P* = 0.020) were independent prognostic factors for patients with gastric cancer (Table 2).

**Table 1:** The correlation between clinicopathological parameters and lncNRA XIST expression levels in 106 gastric cancer patients

Variables	n	High XIST expression (%)	Low XIST expression (%)	*P* value
Age				
< 60	74	35(64.8)	39(75.0)	0.253
≥ 60	32	19(35.2)	13(25.0)	
Gender				
Male	67	33(61.1)	34(65.3)	0.648
Female	39	21(38.9)	18(34.7)	
Tumor size				
< 5 cm	30	10(18.5)	20(38.4)	0.023^a^
≥ 5 cm	76	44(81.5)	32(61.6)	
Peritoneum dissemination				
Absent	89	44(81.4)	45(86.5)	0.478
Present	17	10(18.6)	7(13.5)	
Differentiation				
Well	19	7(12.9)	12(23.0)	0.326
Moderate	36	18(33.3)	18(34.6)	
Poor and others	51	29(43.8)	22(42.4)	
Lymph node invasion				
Absent	31	10(18.5)	21(40.3)	0.013^a^
Present	75	44(81.5)	31(59.7)	
Distant metastasis				
Absent	78	34(62.9)	44(84.6)	0.011^a^
Present	28	20(37.1)	8(15.4)	
TNM stage				
I-II	35	12(22.2)	23(44.2)	0.016^a^
III-IV	71	42(77.8)	29(55.8)	

^a^ *P* **<** 0.05, Chi-square test

**Table 2:** Univariate and multivariate analyses of various potential prognostic factors in 106 gastric cancer patients

Factors	Univariate analysis		Multivariate analysis	
	HR^b^(95 % CI^c^)	*P*	HR^b^(95 % CI^c^)	*P*
Age	1.11(0.92–1.27)	0.238	-	-
Gender	1.25(1.14–1.51)	0.324	-	-
Tumor size	1.45(1.13–1.89)	0.097	-	-
Peritoneum dissemination	0.99(0.73–1.82)	0.549	-	-
Differentiation	1.23(1.11–1.73)	0.095	-	-
Lymph node invasion	1.83(1.25–2.22)	0.057	0.93(0.69–1.44)	0.669
Distant metastasis	2.06(1.47–2.98)	0.018^a^	1.65(1.15–2.56)	0.033^a^
TNM stage	1.66(1.31–2.15)	0.027^a^	1.29(1.08–2.36)	0.253
lncRNA XIST expression	3.11(1.67–3.78)	0.002^a^	1.n1.32–2.26)	0.020^a^

^a^ *P* **<** 0.05

^b^HR, hazard ratio

^c^CI, confidence interval

### Knockdown of lncRNA XIST inhibits cell proliferation, migration and invasion in vitro

The significant increased expression of lncRNA XIST in gastric cancer tissues prompted us to investigate its biological role in gastric cancer cells. Due to the long sequence of lncRNA XIST, we knockdown the expression of lncRNA XIST in gastric cancer cells. Real-time PCR was performed to confirm the successful knockdown of lncRNA XIST in gastric cancer cells (*P < 0.05, Fig. 2b). CCK-8 assay indicated that knockdown of lncRNA XIST significantly inhibited cell proliferation in SGC7901 and AGS cells. (*P < 0.05, Fig. 2c and d). Colony formation assay showed cell transfected with si-XIST formed sifnificantly less clonies than those transfected with si-NC (*P < 0.05, Fig. 2e). Moreover, transwell and wound healing assays demonstrated that the cell invasion and migration was markedly suppressed in gastric cancer cells transfected with si-XIST as compared with cells transfected with si-NC (*P < 0.05, Fig. 2f and g). Besides, we did western blot to test whether XIST can affect epithelial-mesenchymal transition (EMT) in gastric cancer cells. To our interest, knockdown of XIST increased the level of epithelial markers such as E-cadherin, α-catenin while reduced the level of mesenchymal markers such as Vimentin and Fibronectin (Fig. 2h). These data demonstrated that knockdown of lncRNA XIST could inhibit gastric cancer proliferation and invasion in vitro.

### Knockdown of lncRNA XIST inhibit tumor growth and metastasis in vivo

**Fig. 3:**
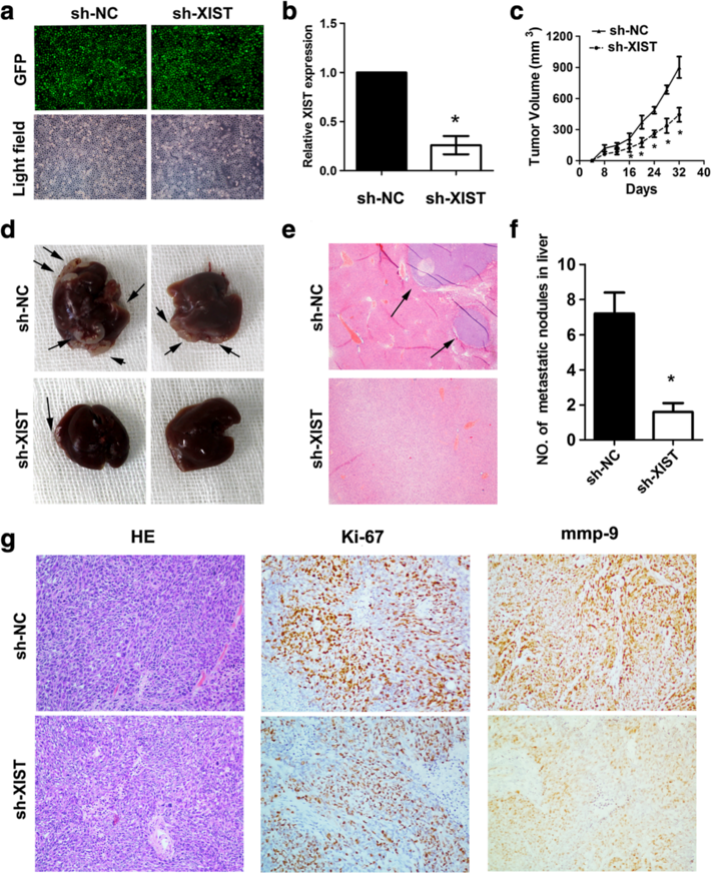
Knockdown of lncRNA XIST expression inhibits tumor growth and metastasis in vivo. **a** and **b** The infection efficiency of lentivirus in SGC7901 cells. Because this vector contains a GFP fragment, the cells emit green fluorescence when infected by the virus. The SGC7901 cells were observed under light and green fluorescence microscopy **(a)**, real-time PCR analysis confirmed the interference efficiency. **c** Knockdown of lncRNA XIST expression significantly inhibited tumor growth of SGC7901 cells, the mean tumor volume was 313 mm^3^ and 858 mm^3^ for the SGC7901/sh-XIST and SGC7901/sh-NC groups, respectively (*P < 0.05). **d**, **e** and **f** Knockdown of lncRNA XIST expression significantly reduced the metastatic nodules in the liver, the mean metastases nodules were 1.7 and 7.3 for the SGC7901/sh-XIST and SGC7901/sh-NC groups, respectively (*P < 0.05)

To explore whether knockdown of lncRNA XIST affects tumor growth and metastasis in vivo. We constructed two stable cell lines by using the lentivirus vector to mediate the knockdown of lncRNA XIST in SGC7901 cells; the resulting cells were designated as SGC7901/sh-XIST and SGC7901/sh-NC cells respectively (Fig. 3a and b). To investigate the in vivo effect of lncRNA XIST knockdown on gastric cancer tumor growth, cells (SGC7901/sh-XIST and SGC7901/sh-NC) were subcutaneously injected into the flank of nude mice. Tumor size was measured every 4 days; after 5 weeks, mice were killed and tumors were dissected out. The results indicated that the volume of tumors formed by SGC7901/sh-XIST cells were significantly smaller than that formed by SGC7901/sh-NC cells, the mean tumor volume was 313 mm^3^ and 858 mm^3^ for the SGC7901/sh-XIST and SGC7901/sh-NC groups, respectively (*P < 0.05, Fig. 3c). To further determine the effect of lncRNA XIST knockdown on tumor metastasis in vivo, SGC7901/sh-XIST and SGC7901/sh-NC cells were transplanted into the lateral tail vein of the nude mice. Six weeks later, mice were killed and liver metastases were examined. In accordance with the in vitro results, the number of metastases to the liver were dramatically reduced in mice injected with SGC7901/sh-XIST cells compared with those of SGC7901/sh-NC cells, the mean metastases nodules were 1.7 and 7.3 for the SGC7901/sh-XIST and SGC7901/sh-NC groups, respectively (*P < 0.05, Fig. 3d, e and f). In addition, we performed IHC to determine whether knockdown of XIST can reduce the expression of Ki-67 and mmp-9 in tissues taken from tumor growth assay in nude mice. The results showed that knockdown of XIST can significantly reduce the level of Ki-67 and mmp-9 (Fig. 3g). These results indicated that knockdown of lncRNA XIST could suppress tumor growth and metastasis in vivo.

### Regulating relationship between lncRNA XIST and miR-101

**Fig. 4:**
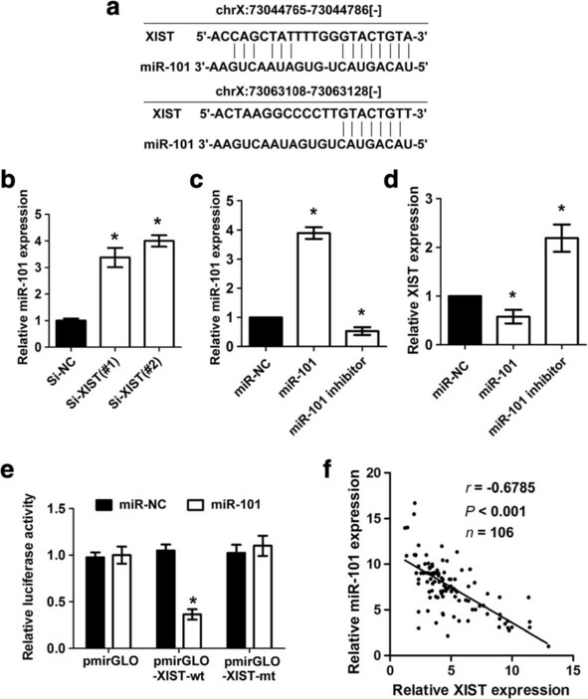
Regulation relationship between lncRNA XIST and miR-101. **a** Schematic representation of the predicted target site for miR-101 in lncRNA XIST. **b** Knockdown of lncRNA XIST increased miR-101 expression in SGC7901 cells (*P < 0.05). **c** Real-time PCR analysis confirmed the miR-101 ectopic expression and miR-101 inhibition in SGC7901 cells (*P < 0.05). **d** Ectopic miR-101 expression decreased lncRNA XIST expression while inhibition of miR-101 increased lncRNA XIST expression (*P < 0.05). **e** Luciferase reporter assay in human embryonic kidney (HEK) 293 T cells, co-transfected with the reporter plasmid (or the corresponding mutant reporter) and the indicated miRNAs. miR-101 significantly decreased the luciferase activity in XIST-wt but not in XIST-mt (*P < 0.05). **f** The expression of lncRNA XIST was inversely correlated with the expression level of miR-101 in gastric cancer tissues (*P < 0.05)

Increasing evidences showed that lncRNAs can function as competitive endogenous RNA (ceRNA) for miRNAs or naturally occurring miRNA sponges. Such ceRNA networks have identified as critical regulators of gene expression and signaling pathways. We first used the bio-informatic tools to search for the potential miRNAs that can be regulated by lncRNA XIST, to our interest, miR-101, which has been found to be down-regulated in gastric cancer, could bind to lncRNA XIST. Fig. 4a showed the binding sites between lncRNA XIST and miR-101. We detected the expression of miR-101 in SGC7901 cells after knockdown of lncRNA XIST, the result showed that miR-101 level was significantly increased in SGC7901/sh-XIST cells than that of SGC7901/sh-NC cells (*P < 0.05, Fig. 4b). Then, we evaluated whether miR-101 can regulate lncRNA XIST expression by determine the effects of miR-101 ectopic expression and inhibition on the expression of lncRNA XIST. As shown in Fig. 4c and d, lncRNA XIST expression was decreased after ectopic expression of miR-101, whereas increased after inhibition of miR-101 (*P < 0.05). To confirm the direct binding relationship between lncRNA XIST and miR-101, a luciferase activity assay was conducted. The predicted miR-101 binding site (XIST-wt) and its mutant type (XIST-mt) were amplified and directly fused to the downstream of the luciferase reporter gene in the pmirGLO-basic vector. Co-transfetion of miR-101 and pmirGLO-XIST-wt significantly decreased the luciferase activity, whereas co-transfection of miR-NC and pmirGLO-XIST-wt did not change the luciferace activity (*P < 0.05, Fig. 4e). Likewise, cells co-transfected with miR-101 and pmirGLO-XIST-mt showed no obvious change in luciferase activity (*P < 0.05, Fig. 4e). To investigate whether there was inverse correlation between lncRNA XIST and miR-101 in gastric cancer tissues, we conducted RT-PCR in 106 gastric tissues to measure the level of lncRNA XIST and miR-101, a significant inverse correlation was found between lncRNA XIST levels and miR-101 levels in the gastric cancer tissues (*r* = −0.6785, *P* < 0.001, Fig. 4f). These results demonstrated that there exist a negative regulation between lncRNA XIST and miR-101.

### lncRNA XIST regulates miR-101 to modulate EZH2 in gastric cancer cells

**Fig. 5:**
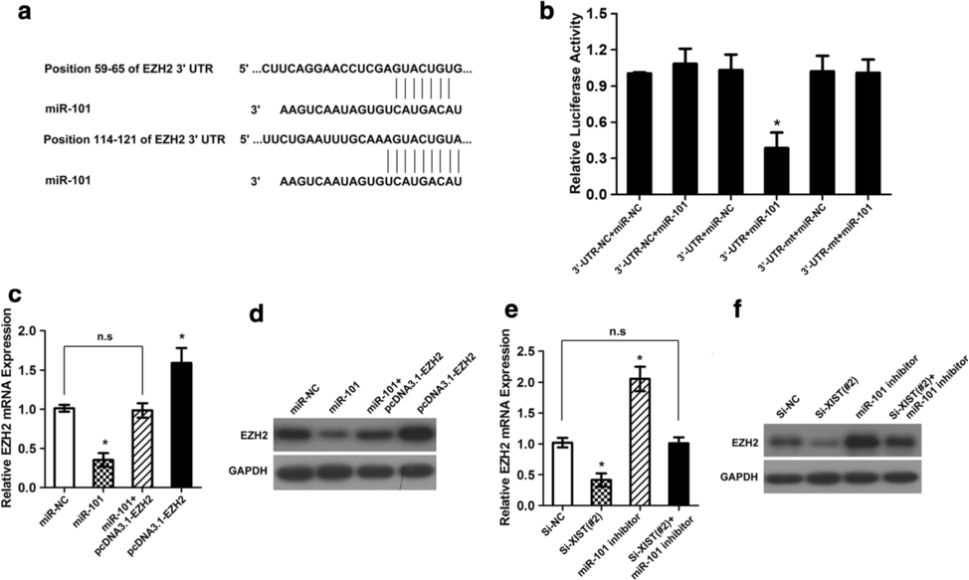
lncRNA XIST regulates EZH2 expression by acting as a molecular sponge. **a** The predicted miR-101 binding sites in EZH2 mRNA 3′-UTR as predicted by Targetscan algorithm. **b** Luciferase reporter assay for EZH2 mRNA 3′-UTR following miR-101 ectopic expression (*P < 0.05). **c** and **d** EZH2 mRNA and protein level in SGC7901 cells following ectopic expression of miR-101 and/or EZH2 expression vector lacking the 3′-UTR (*P < 0.05). **e** and **f** EZH2 mRNA and protein level in SGC7901 cells following knockdown of lncRNA XIST and/or inhibition of miR-101 (*P < 0.05)

**Fig. 6:**
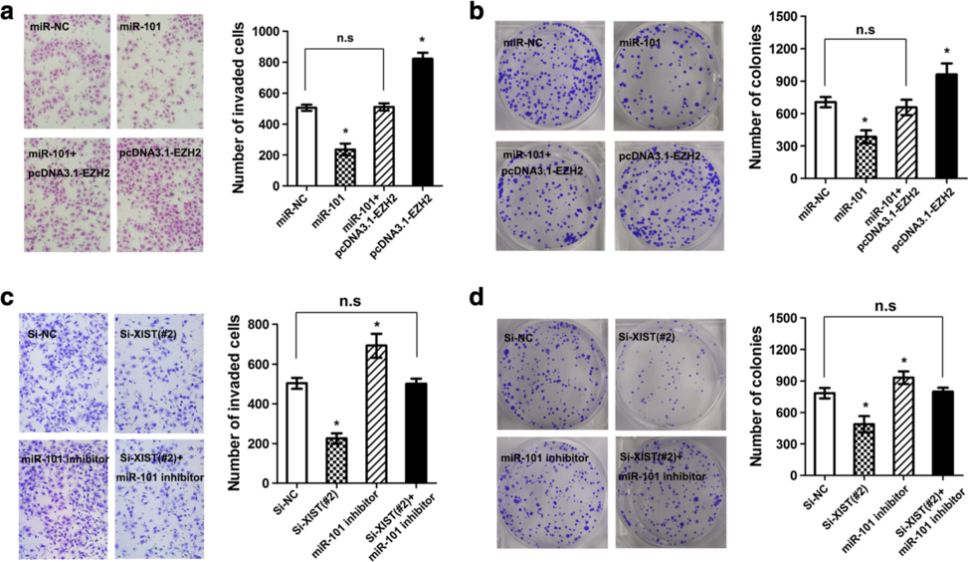
EZH2 expression mediated the biological effects exerted by lncRNA XIST. **a** and **b** Cell invasion and colony formation assay following ectopic expression of miR-101 and/or EZH2 expression vector lacking the 3′-UTR (*P < 0.05). **c** and **d** Cell invasion and colony formation assay following knockdown of lncRNA XIST and/or inhibition of miR-101 (*P < 0.05)

Having demonstrated that lncRNA XIST can affect miR-101 expression, we considered the functional aspect. It is known that miRNAs function by targeting downstream genes, and EZH2 has been proved to be involved in gastric cancer progression. Firstly, bioinformatic tools confirmed the potential miR-101 binding sites in the 3′-UTR of EZH2 (Fig. 5a). miR-101 was able to markedly reduce the relative luciferase activity of EZH2 3′UTR-wt in the SGC7901 cells (*P < 0.05), whereas that in the cells transfected with EZH2 3′UTR-mt was not decreased (Fig. 5b). Moreover, the mRNA and protein levels of EZH2 were significantly decreased by miR-101 ectopic expression in both the SGC7901 cells, and this reduction could be restored by co-transfected with a pcDNA3.1-EZH2 vector, which contained the coding sequences but lacked the 3′-UTR of EZH2 (*P < 0.05, Fig. 5c and d). Then, we explore whether lncRNA XIST can modulate the expression of EZH2, to our interest, we found that knockdown of lncRNA XIST was able to significantly reduce the mRNA and protein levels of EZH2, what’s more, knockdown of lncRNA XIST could reduce the mRNA and protein levels which was increased by miR-101 inhibitor (*P < 0.05, Fig. 5e and f). In addition, ectopic expression of miR-101 inhibited the colony formation and invasion ability of SCG7901 cells, and the suppressive effects could be conteracted by co-transfected with a pcDNA3.1-EZH2 vector, which contained the coding sequences but lacked the 3′-UTR of EZH2 (*P < 0.05, Fig. 6a and b). Likewise, knockdown of lncRNA XIST could suppress the colony formation and invasion ability stimulated by miR-101 inhibition in SGC7901 cells (*P < 0.05, Fig. 6c and d). These results indicated that lncRNA XIST can regulates miR-101 to modulate EZH2 in gastric cancer cells.

## Discussion

Recent studies have indicated that lncRNAs play critical roles in cancer development and metastasis [28, 29]. Increasing reports revealed that lncRNA expression was significantly altered in gastric cancer tissue through screening lncRNA expression profile. For example, Yang et al. found that lncRNA H19 was up-regulated in tumor tissues and cells as compared with normal controls, moreover, forced expression of H19 promoted cell growth [30]. Sun et al. reported that the expression of GAS5 was significantly down-regulated in gastric cancer, and low expression of GAS5 was associated with adverse disease-free survival and overall survival of patients with gastric cancer. In addition, overexpression of GAS5 could inhibit gastric cancer cell proliferation and induce apoptosis both in vitro and in vivo [31]. More recently, Huang et al. indicated lncRNA KRT7-AS is activated in gastric cancer and promotes cancer progression by increasing KRT7 expression [32].

In our study, we found the expression level of lncRNA XIST was significantly higher in gastric cancer tissues than that of adjacnet normal tissues. Higher expression of lncRNA XIST was positively associated with larger tumor size, lymph node invasion, distant metastasis and TNM stage. In addition, overexpression of lncRNA XIST was associated with adverse prognosis and could be used as an independent prognostic factor. These results implicated that lncRNA XIST may play an important role in gastric cancer progression. Consistent with our results, previous studies have found that lncRNA XIST expression was up-regulated in patient with collecting duct carcinoma of the kidney, sporadic human colorectal cancer, and gastric fundus of a male mouse infected with helicobacter felis which can lead to gastric cancer [33–35]. However, some other studies indicated that lncRNA XIST was lost in breast, cervical and ovarian cancer cell lines [36, 37]. These results demonstrated that lncRNA XIST exhibit remarkably tissue-specific expression patterns, and it can play oncogenic or tumor-suppressive role depending on the cancer type and cellular context.

Previous reports have revealed that lncRNA XIST was essential for long term survival of hematopoietic stem cells [19], suppression of lncRNA XIST could markedly impair the early phase of female pluripotent stem cells induction [38], and knockdown of lncRNA XIST exerts tumor-suppressive functions in human glioblastoma stem cells [20]. In our study, we found that knockdown of lncRNA XIST inhibited cell proliferation, migration and invasion in vitro. Moreover, in vivo study also confirmed that knockdown of lncRNA XIST could suppress tumor growth and distant metastasis. These results indicated that dysregulation of lncRNA XIST might be a common incidence in different tumors.

Mounting reports found that lncRNAs can funtion as ceRNA for miRNAs, they act as molecular sponges to competitively inhibit miRNAs. For example, lncRNA GAS5 can function as a ceRNA for miR-21 [39]; long non-coding RNA H19 promotes glioma cell invasion by deriving miR-675 [40]; Long non-coding RNA MEG3 functions as a competing endogenous RNA of miR-181 s to regulate gastric cancer progression [41]. We speculated that lncRNA XIST might be a ceRNA in gastric cancer. Using bioinformatics databases (Starbase v2.0, miRcode and RNAhybrid), we identified 23 miRNAs which may interact with lncRNA XIST. Further studies indicated that only miR-101 expression was significantly increased upon lncRNA XIST knockdown. On the contrary, ectopic expression of miR-101 reduced expression of lncRNA XIST, whereas inhibition of miR-101 inreased lncRNA XIST expression. These data suggested that an inverse correlation existed between lncRNA XIST and miR-101. In addition, a luciferase activity assay confirmed the direct binding relationship bwtween lncRNA XIST and miR-101. A recent study demonstrated that lncRNA XIST fuctions as a ceRNA for miR-152 in glioma [20]. This implying that lncRNA XIST may interact with different miRNAs in different tumors.

miR-101 has been reported to be down-regulated and in different tumors [42, 43]. In gastric cancer, previous studies have demonstrated that down-regulation of miR-101 resulted in overexpression of EZH2 and promoted tumor progression [26, 44]. This prompted us to investigate whether knockdown of lncRNA XIST exerted its tumor-suppressive effects through regulating of EZH2 expression. We first confirmed that miR-101 could negatively regulate EZH2 expression in gastric cancer cells. Furthermore, knockdown of lncRNA XIST was able to reduce the mRNA and protein levels of EZH2, what’s more, knockdown of lncRNA XIST could reduce the mRNA and protein levels of EZH2 which was increased by miR-101 inhibitor. In addition, knockdown of lncRNA XIST could suppress the colony formation and invasion ability stimulated by miR-101 in gastric cancer cells. These data revealed that lncRNA XIST exerts its biological effects at least in part by modulating EZH2 expression. This is in accordance with our previous results that EZH2 is overexpressed and promotes tumor progression in gastric cancer [45].

## Conclusions

In this study, we demonstrated that lncRNA XIST was significantly overexpressed in gastric cancer tissues and cell lines. Overexpression of lncRNA XIST was closely associated with an aggressive tumor phenotype and adverse prognosis in gastric cancer patients. Knockdown of lncRNA XIST suppressed cell proliferation, migration and invasion in vitro and tumorigenesis and metastasis in vivo. Furthermove, an inverse relationship was found between lncRNA XIST and miR-101, and knockdown of lncRNA XIST exerted its tumor-suppressive effects at least in part through regulating miR-101 to modulate EZH2 expression. Therefore, we provided the first evidence that knockdown of lncRNA XIST could inhibit gastric cancer progression and metastasis by modulating the miR-101/EZH2 pathway.

## Abbreviations

3′-UTR: 3′-untranslated regions
CCK-8: Cell counting kit-8
EZH2: X-inactive specific transcript
FBS: Fetal bovine serum
lncRNA: Long non-coding RNA
miR-101: microRNA-101
shRNA: Short hairpin RNA
siRNA: Small interfering RNA
TNM: Tumor, lymph node, metastasis
XIST: X-inactive specific transcript
