# Correction to: Long non-coding RNA XIST regulates gastric cancer progression by acting as a molecular sponge of miR-101 to modulate EZH2 expression

**DOI:** 10.1186/s13046-021-02002-9

**Published:** 2021-06-24

**Authors:** Dong-liang Chen, Huai-qiang Ju, Yun-xin Lu, Le-zong Chen, Zhao-lei Zeng, Dong-sheng Zhang, Hui-yan Luo, Feng Wang, Miao-zhen Qiu, De-shen Wang, Da-zhi Xu, Zhi-wei Zhou, Helene Pelicano, Peng Huang, Dan Xie, Feng-hua Wang, Yu-hong Li, Rui-hua Xu

**Affiliations:** 1 State Key Laboratory of Oncology in South China, Collaborative Innovation Center for Cancer Medicine, Sun Yat-sen University Cancer Center, Guangzhou, PR China; 2 University of Texas M.D. Anderson Cancer Center, Houston, TX USA

**Correction to: J Exp Clin Cancer Res 35, 142 (2016)**

**https://doi.org/10.1186/s13046-016-0420-1**

Following the publication of the original article [1], the authors identified that mismatched images had been used in Fig. 6; specifically the panels “Si-XIST(#2)+miR-101 inhibitor” in Fig. 6c. The corrected figure is given below. The correction does not affect the conclusion of the article. The original article has been corrected.

**Fig. 6:**
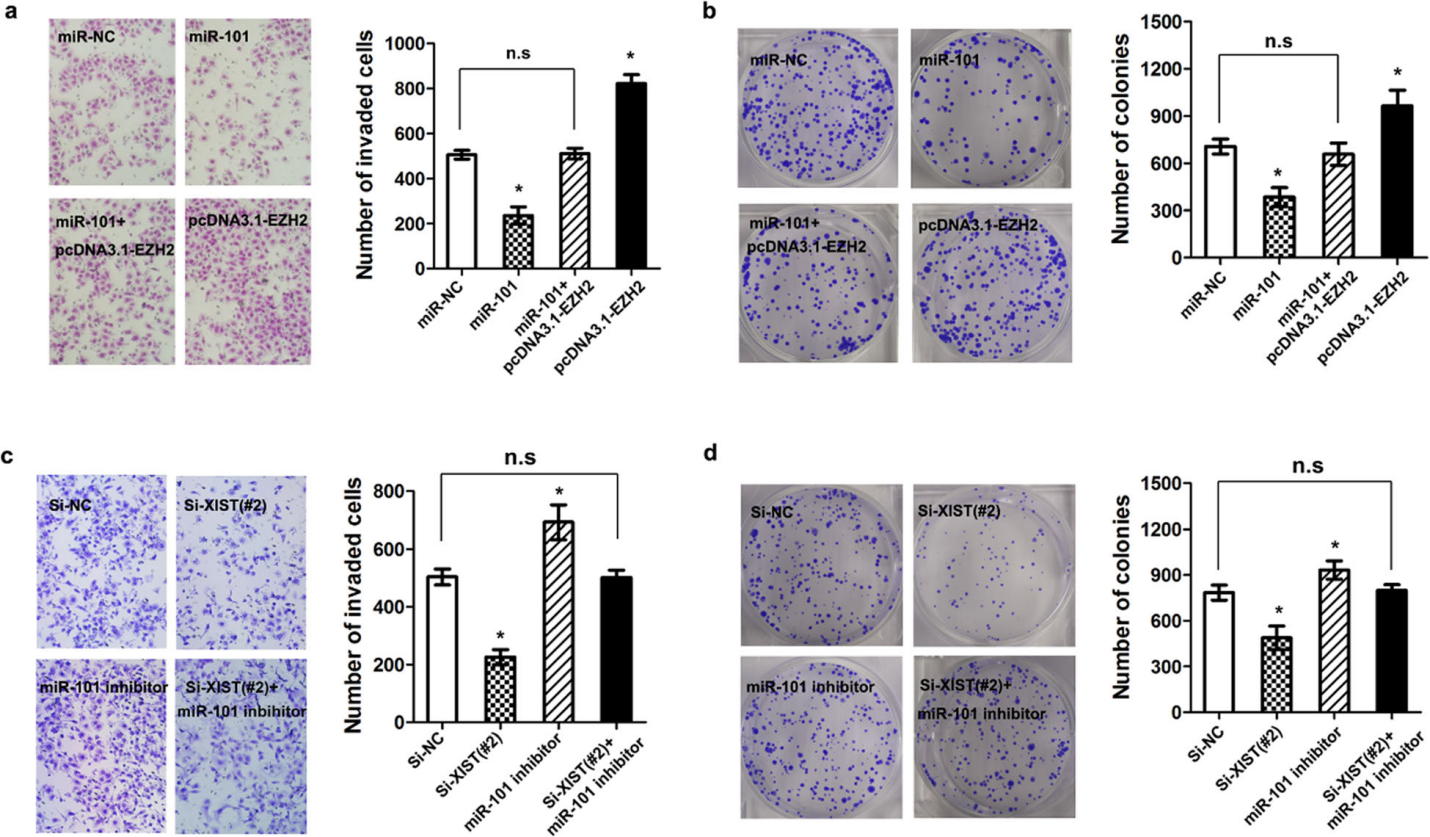
EZH2 expression mediated the biological effects exerted by lncRNA XIST. **a** and **b** Cell invasion and colony formation assay following ectopic expression of miR-101 and/or EZH2 expression vector lacking the 3’-UTR (*P < 0.05). **c** and **d** Cell invasion and colony formation assay following knockdown of lncRNA XIST and/or inhibition of miR-101 (*P < 0.05)
